# Macrocycle-Antibiotic Hybrids: A Path to Clinical Candidates

**DOI:** 10.3389/fchem.2021.659845

**Published:** 2021-04-30

**Authors:** Abdrrahman Shemsu Surur, Dianqing Sun

**Affiliations:** Department of Pharmaceutical Sciences, The Daniel K. Inouye College of Pharmacy, University of Hawai‘i at Hilo, Hilo, HI, United States

**Keywords:** antibiotic hybrid, macrocycle, macrocycle hybrid, TD-1792, TD-1607, TNP-2092, TNP-2198, DSTA4637S

## Abstract

The tale of abate in antibiotics continued defense mechanisms that chaperone the rise of drug-defying superbugs—on the other hand, the astray in antibacterial drug discovery and development. Our salvation lies in circumventing the genesis of resistance. Considering the competitive advantages of antibacterial chemotherapeutic agents equipped with multiple warheads against resistance, the development of hybrids has rejuvenated. The adoption of antibiotic hybrid paradigm to macrocycles has advanced novel chemical entities to clinical trials. The multi-targeted TD-1792, for instance, retained potent antibacterial activities against multiple strains that are resistant to its constituent, vancomycin. Moreover, the antibiotic conjugation of rifamycins has provided hybrid clinical candidates with desirable efficacy and safety profiles. In 2020, the U.S. FDA has granted an orphan drug designation to TNP-2092, a conjugate of rifamycin and fluoroquinolone, for the treatment of prosthetic joint infections. DSTA4637S is a pioneer antibacterial agent under clinical development and represents a novel class of bacterial therapy, that is, antibody–antibiotic conjugates. DSTA4637S is effective against the notorious persistent *S. aureus* bacteremia, a revelation of the abracadabra potential of antibiotic hybrid approaches.

## Introduction

Relentlessness in developing resistance is imperiling the competence of the antibacterial arsenal. A global health crisis is heightened by the emergence and prevalence of multidrug-resistant bacteria pathogens, which are untreatable with commonly used antibiotics (Aslam et al., 2018). According to the landmark 2019 Centers for Disease Control and Prevention (CDC) estimate, there are over 2.8 million antibiotic-resistant infections every year in the United States, which account for a death every 15 min (CDC, 2019). The once though magic bullets are no longer producing magical chemotherapeutic effects. The rising and compounding antimicrobial resistances continue to mandate novel strategies in the antibacterial drug discovery and development process (Silver, 2011; Brown and Wright, 2016; Shang et al., 2020). The 2019 analysis of antibacterial agents in clinical development by the World Health Organization (WHO) indicates the current clinical pipeline is insufficient to alleviate the threats posed by antimicrobial resistance (WHO, 2019a).

## Antibiotic Combinations Versus Antibiotic Hybrids

Single-target agents have dominated the current antibacterial collection, and bacteria seem capable of rendering all ineffective (East and Silver, 2013). Simultaneous use of drug molecules having different molecular targets, or polypharmacology, was then considered more reliable to eliminate or at least slow the onset of resistance (Gray and Wenzel, 2020). Accordingly, concomitant use of antibiotics is often practiced by clinicians to prevent the development of resistance, broaden the spectrum of activity, and/or optimize the dose of drugs. In combination therapy, an adjuvant which may be inactive on its own is used with an antibiotic—a strategy called antibiotic–adjuvant approach (Liu et al., 2019). The adjuvant may enable entrance of the bona fide antibiotic by improving the membrane permeability, inhibit enzymes responsible for inactivation, or prevent the active efflux of the antibiotic (Liu et al., 2019; Tyers and Wright, 2019). Antibiotic–antibiotic combination approach, on the other hand, entails the use of dynamic dual antibiotics to achieve drug synergism or suppress the development of resistance, hypothesizing that bacteria will not survive the one-two punch of the antibiotics (Tyers and Wright, 2019). This antibiotic cocktail approach has prolonged the clinical utilities of some antibiotics, albeit potential problems due to unfavorable pharmacokinetic (PK) interactions, and for several reasons, the boosted *in vitro* activity often does not translate well into *in vivo* efficacy in animal models or clinical settings (Klahn and Bronstrup, 2017; Domalaon et al., 2018). In a scenario where the combined drugs lack PK complementarities, such as dissimilar half-lives where a short-acting drug is excreted rapidly, or different tissue distributions where one component is barely distributed, the other component will become vulnerable from the aspect of development of resistance (Domalaon et al., 2018). For example, multiple resistance mechanisms have gradually limited the clinical use of co-trimoxazole involving the dihydrofolate reductase (DHFR) inhibitor trimethoprim and the dihydropteroate synthase (DHPS) inhibitor sulfamethoxazole, the esteemed example of the antibiotic combination approach (Eliopoulos and Huovinen, 2001). Pharmacokinetic disparities, such as dissimilar volume of distributions by the virtue of differences in hydrophobic properties between trimethoprim and sulfamethoxazole, might have contributed to the development of resistance (Brown, 2014). In addition, drug combination or co-formulation is also vulnerable to additive toxicities (Tamma et al., 2012). The need for taking multiple drugs, especially if different routes of administrations are involved, may also deflate patients’ convenience (Fisher et al., 2020).

An attractive alternative to the mix and match antibiotic combinations is to bridge two pharmacophores by a metabolically stable covalent bond to generate a heteromeric synthetic construct, which behaves as a single chemical entity pertaining to PK parameters, a strategy otherwise known as an antibiotic hybrid (Pokrovskaya and Baasov, 2010; Klahn and Bronstrup, 2017). Diverse subjective definitions are forged for antibiotic hybrids. Generally, the antibiotic hybrid umbrella covers dual-acting antibiotic hybrids, divalent or multivalent antibiotics, antibiotic conjugates, chimeric antibiotics, and antibiotic hybrid prodrugs (Wang et al., 2016; Domalaon et al., 2018). The latter involves a cleavable linker between synthons, which can be metabolized only by a specific strain, a very useful strategy for the development of narrow-spectrum antibacterial agents (Domalaon et al., 2018; Jubeh et al., 2020). In the belligerence of drug resistance, molecules composed of two or more active structure motifs that are capable of acting at their respective targets have been extensively explored (Shavit et al., 2017). Selection of a matching partner in the antibiotic hybrid strategy is crucial so that the resulting dual-acting hybrid will unlikely suffer from cross-resistance. An organism may develop resistance to a dual-acting hybrid if it is not susceptible to the action of either drug (Parkes and Yule, 2016).

Over the past few decades, significant advances have been made in medicinal chemistry and chemical biology of macrocyclic compounds (Yu and Sun, 2013; Yudin, 2015). In contrast to large macromolecules and small synthetic molecules, macrocycles possess unique structural advantages and benefits from featuring both large molecules such as high potency and impeccable selectivity, and small molecules such as reasonable manufacturing costs, favorable PK, and the lack of immunogenicity (Peterson, 2017). The up-to-date comprehensive review of antibacterial agents in clinical pipeline (Butler and Paterson, 2020) along with reviews regarding the 14- to 15-membered macrolide hybrids (Janas and Przybylski, 2019) and their potential as anti-infective and anti-inflammatory agents (Paljetak et al., 2017) indicates the antibiotic hybrid approach is trending in the development of macrocyclic compounds. In this review, using terms such as “drugs” and “bacterial infections,” clinical trials listed in clinicaltrials.gov were searched meticulously. More than 2,000 clinical trials matched our search criteria, and the search results were cross-checked with the 2019 global observatory of the WHO for antibacterial products in clinical development (WHO, 2019b). Five novel macrocycle-based antibiotic hybrids under clinical developments, that is, TD-1792, TD-1607, TNP-2092, TNP-2198, and DSTA3647S, are highlighted and discussed.

## Hybrids of Vancomycin

**FIGURE 1:**
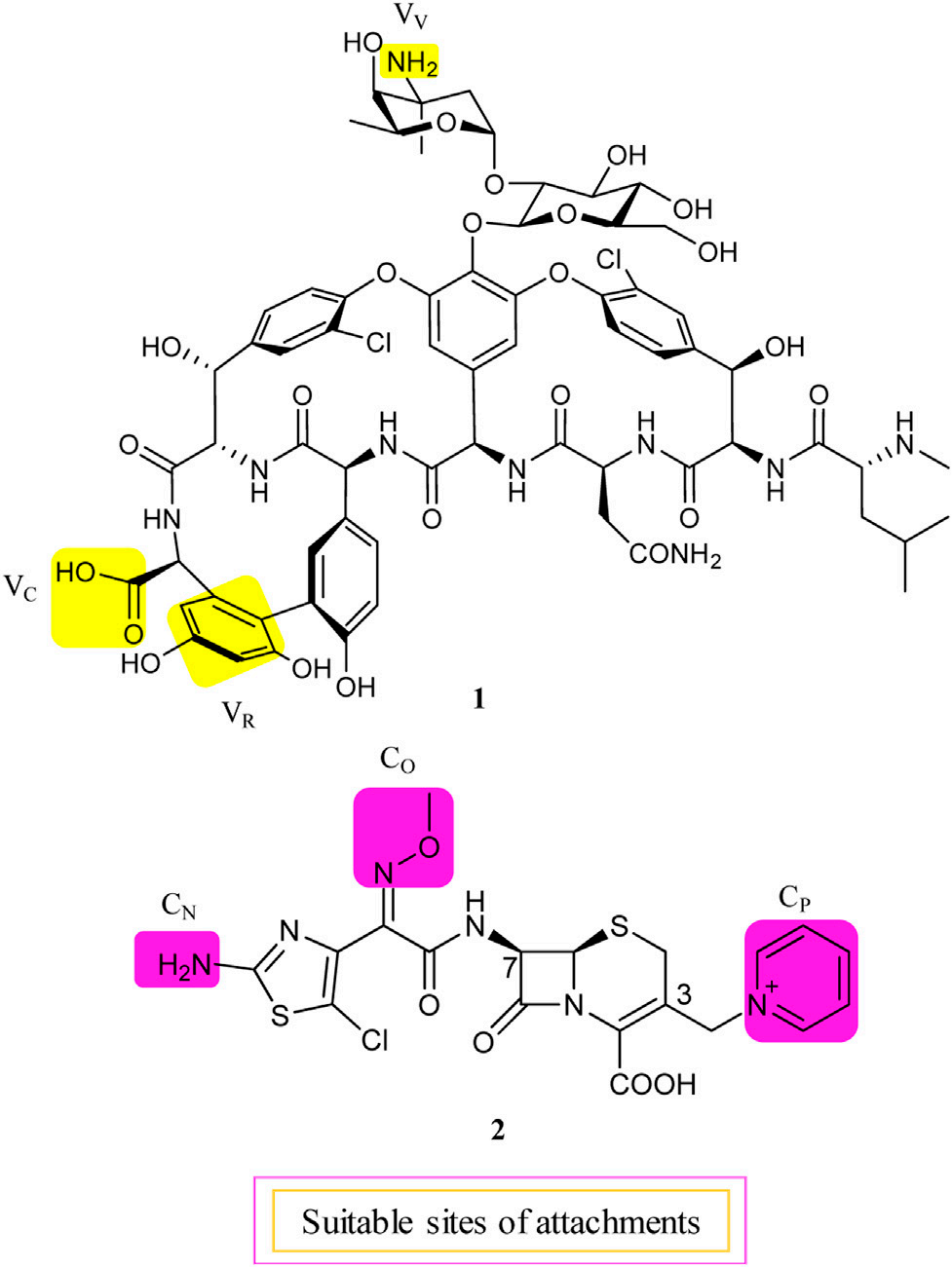
Suitable attachment positions in vancomycin (**1**) and THRX-169797 (**2**).

**FIGURE 2:**
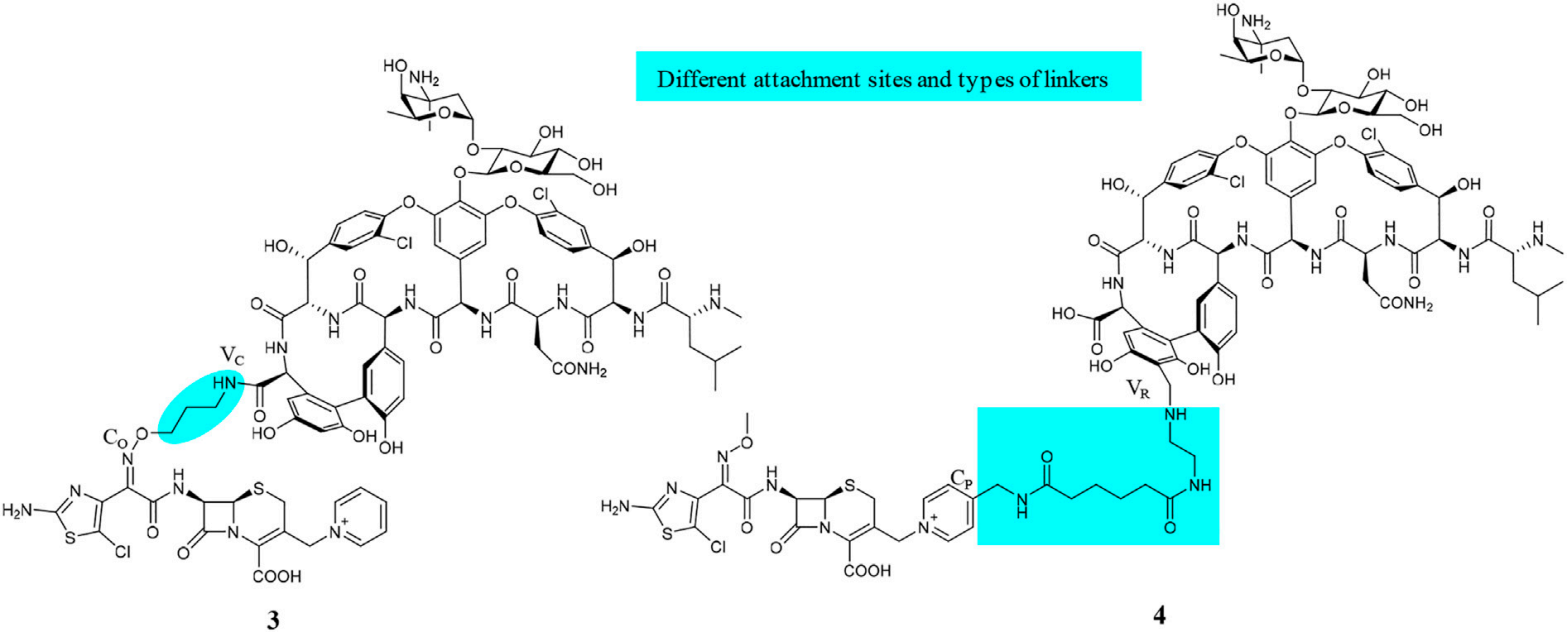
Chemical structures of VAN hybrids, cefilavancin (**3**, TD-1792), and TD-1607 (**4**).

Although about 6 decades have passed since vancomycin (VAN, **1**, Figure 1) was initially approved by the U.S. Food and Drug Administration (FDA) in 1958 and introduced into clinical practice, it is still commonly used for the treatment of many Gram-positive bacterial infections and often the last resort in modern treatment of drug-resistant infections (Okano et al., 2017). However, a dark cloud lids over the use of **1**, as evidenced by widespread VAN-resistant *Enterococci* (VRE) and VAN-resistant *Staphylococcus aureus* (VRSA) and the loss of clinical efficacy against severe methicillin-resistant *S. aureus* (MRSA) infections (Faron et al., 2016). Vancomycin has continued to be captured in the spotlight in antibacterial drug development; its unique clinical successes, absence of cross-resistance with other antibacterial classes, a significant time span (30 years) between discovery and appearance of the first resistant strains in VRE, and advances in structural determinations or synthetic methods are among the reasons accounted (Blaskovich et al., 2018). Notably, three new structural analogs, that is, telavancin (Vibativ®), dalbavancin (Dalvance®), and oritavancin (Orbactiv®), were approved by the U.S. FDA in September 2009, May 2014, and August 2014, respectively, for the treatments of complicated skin and skin structure infections (cSSSIs) and acute bacterial skin and skin structure infections (ABSSSIs) (Lexicomp, 2021). Besides its historical clinical success and impact, **1** has also been targeted in the antibiotic hybrid paradigm for two main reasons. First, the glycopeptide scaffold in **1** provides several substituents suitable for binding a partner—in particular, the free C-terminal carboxylic acid (V_C_), the primary amine in vancosamine sugar (V_V_), and the aryl group of the seventh amino acid (V_R_) (Figure 1, Long et al., 2008a; Long et al., 2008b; Blaskovich et al., 2018). Second, **1** increases the affinity to the target *via* cooperative back-to-back dimerization. This opens a characteristic possibility to design potent conjugates where two vancomycin residues are dimerized or **1** is covalently linked to another partner such as **2** (Blaskovich et al., 2018). Although **1** has been dimerized or conjugated with siderophores or fluorophores, only its antibiotic hybrids have provided advanced candidates under clinical development, that is, cefilavancin (**3**, also known as TD-1792) and TD-1607 (**4**) in Figure 2 (Negash et al., 2019; Theuretzbacher, 2020).

The lipid intermediate II (a membrane-anchored cell wall precursor) and transpeptidase (the penicillin-binding protein), the cellular targets of **1** and cephalosporins, respectively, are in close proximity and catalyze sequential roles in the bacterial cell wall biosynthesis. Consequently, their hybrid molecule might be able to inhibit both targets simultaneously and therefore have superior bactericidal properties (Long et al., 2008a). As mentioned above, **1** is equipped with at least three potential attachment sites (V_V_, V_C_, and V_R_) (Long et al., 2008a; Blaskovich et al., 2018). Similarly, the C-3 pyridinium (C_P_) moiety, the *Z*-oriented oxime (C_O_), and thiazolylamino (C_N_) groups of the C-7 side chain of 2 are suitable attachment sites in a cephalosporin nucleus (Figure 1, Long et al., 2008a). All possible nine hybrid heteromers between the above-mentioned attachment sites were synthesized *via* an amide linker and exhibited excellent activity against a select panel of Gram-positive bacteria pathogens, including MRSA and vancomycin intermediate-resistant *S. aureus* (VISA) (Long et al., 2008a). Cefilavancin was among the two most promising heteromers, with an MIC_90_ value of 0.03 μg/ml against MRSA isolates (Long et al., 2008a; Blais et al., 2012). Furthermore, *in vitro* studies revealed that THRX-169797 (**2**), the cephalosporin constituent of **3**, was much less active than **3** against all *S. aureus* isolates tested (Blais et al., 2012). The murine neutropenic thigh infection model of MRSA was extended to **3**. The ED_50_ of 0.19 mg/kg for **3** was 40 times more effective than the ED_50_ of that for **1**, with an ED_50_ of 8.1 mg/kg in their assay (Long et al., 2008a). Consequently, **3** was selected and advanced as a clinical candidate for further development.

Cefilavancin also showed very potent activity against the heterogeneous VISA (hVISA), with minimum inhibitory concentration (MIC_90_) values of 0.03 μg/ml against 39 isolated strains of hVISA, relative to 2 μg/ml for **1** (Blais et al., 2012). In addition, **3** was the most active (MIC_90_ = 0.125 μg/ml) against various clinical isolates of VAN-intermediate staphylococcal species (VISS), heterogeneous VISS (hVISS), and VRSA, compared to daptomycin, quinupristin–dalfopristin, and linezolid (Leuthner et al., 2010). A similar study identified the effect of plasma proteins on the potency of **3** as low, and the MIC_90_ value remained the lowest compared to all comparators (Leuthner et al., 2010). Moreover, the positive results from clinical trials have supported the continued development of **3**. In phase 1 studies, plasma concentrations after intravenous (IV) administration of 2 mg/kg body weight were continuously above the MIC value at which 100% MRSA isolates were inhibited (Blais et al., 2012). In phase 2 studies, the efficacy of **3** was compared with that of **1** in the treatment of cSSSIs. The cure rate at the end of the treatments with **1** and **3** was 90.7% and 91.7%, respectively (Stryjewski et al., 2012). The incidence of the most common adverse effects was similar in both groups, except for itching, which was more common in volunteers in the VAN arm (NCT00442832, 2006; Stryjewski et al., 2012). Currently, Theravance Biopharma and R-Pharm are conducting a phase 3 clinical development of **3** in Russia (WHO, 2019a; Adis Insight, 2020).

The hybridization of the nuclei in **3**, but with a longer amide linker and different attachment positions, led to TD-1607 (**4**) (Figure 2, Fatheree et al., 2005). In **4**, the aryl group closest to the C-terminus of **1** (V_R_) is aminomethylated and linked to the pyridinyl substituent (C_P_) from the bicyclic lactam of cephalosporin (Fatheree et al., 2005; Blaskovich et al., 2018). TD-1607 exerts its antibacterial activity by inhibiting cell wall biosynthesis; *in vitro* microbiological profiling showed a rapid and potent bactericidal effect against Gram-positive organisms (Sader et al., 2014). Compound **4** proved to be the most effective against all comparators, including **1**, with MIC values between 0.008 and 0.06 μg/ml against 1,026 MRSA isolates procured worldwide (Sader et al., 2014; Liapikou et al., 2017). Single and multiple ascending dose phase 1 studies on the safety, tolerability, and PK of **4** were conducted in healthy volunteers (NCT01791049, 2013; NCT01949103, 2013). However, Theravance BioPharma has discontinued further clinical developments of **4** (Butler and Paterson, 2020).

### Synthesis of Vancomycin Hybrids

**SCHEME 1:**
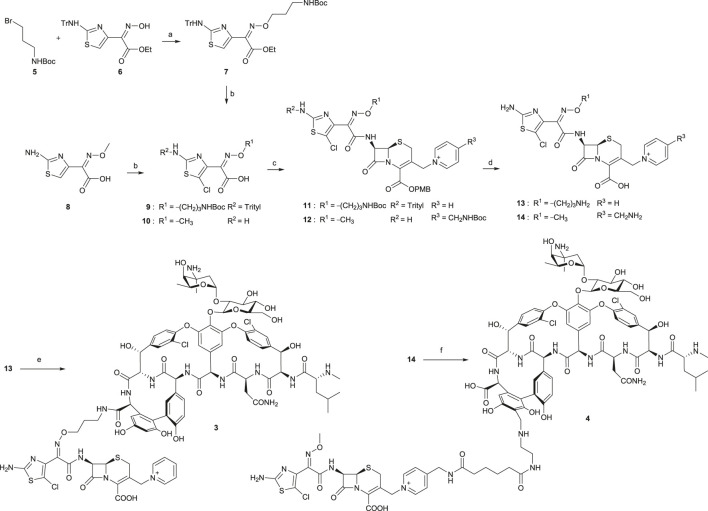
Synthesis of VAN hybrids **3** and **4** (Long et al., 2008a). **(a)** Cs_2_CO_3_, tetrabutylammonium iodide, DMF, rt, 2 h. **(b)** for **9**: i. KOH, ethanol, 80°C, 30 min; ii. NCS, CHCl_3_, rt, overnight; for **10**: NCS, DMF, rt, overnight. **(c)** for **11**: i. ACLE, THF, –45°C, 2,4,6-collidine, POCl_3_, 10 min; ii. NaI, acetone, N_2_, rt, 80 min, then pyridine, 150 min; for **12**: i. ACLE, *N*-[3-dimethylaminopropyl]-*N*′-ethylcarbodiimide hydrochloride (EDCI), 2,4,6-collidine, DMF, rt, 2 h; ii. Acetone, NaI, N_2_, 4-(*N*-*tert*-butoxycarbonyl)aminomethyl pyridine, rt, 2 h. **(d)** TFA, dichloromethane, anisole, rt, 2–3 h. **(e) 1**, benzotriazol-1-yl-oxytripyrrolidinophosphonium hexafluorophosphate (PyBOP), HOAt, DIPEA, DMSO, DMF, rt, 30 min, then 2,4,6-collidine, 4 h. **(f)** di-HOAt adipic ester, DMF, ice bath, 2,4,6-collidine, 15 min, then the V_R_ synthon of **1**, DMF, ice bath, 2,4,6-collidine, 20 min.

Chemical syntheses of VAN conjugates **3** and **4** involved a series of steps to construct the cephalosporin synthon attached to a linker and an eventual coupling with vancomycin (Scheme 1). The synthesis of a cephalosporin and an amide anchor motif in **3** started by coupling *N*-Boc–protected bromopropylamine **5** with the hydroxylimino moiety of a trityl-protected aminothiazolyl residue **6** (Long et al., 2008a). Following ester hydrolysis of **7** and subsequent chlorination of the thiazolyl ring in **7** or **8** using *N*-chlorosuccinimide (NCS) yielded **9** or **10**, respectively. Intermediates **9** and **10** were next coupled with 7-amino-3-chloromethyl-3-cephem-4-carboxylic acid *p*-methoxybenzyl ester hydrochloride salt (ACLE). The 3-chloro good leaving group in the ACLE moiety was replaced with pyridine or 4-(*N*-*tert*-butoxycarbonyl)aminomethyl pyridine to give **11** or **12**, respectively (Fatheree et al., 2005; Long et al., 2008a). The key cephalosporin intermediates **13** and **14** were synthesized by global deprotection of their corresponding precursors in TFA. Finally, VAN **1** was attached directly to **13** through amide bond formation to afford target compound **3** (Fatheree et al., 2005; Long et al., 2008a). Unlike the direct coupling in **3**, target compound **4** was synthesized *via* sequential bifunctional amide coupling of the cephalosporin C_P_ synthon **14** and the VAN V_R_ synthon *via* the di-HOAt ester of adipic acid (Fatheree et al., 2005; Long et al., 2008a).

## Hybrids of Rifamycins

Various clinical guidelines for the treatment of staphylococcal prosthetic joint infection (PJI), including the Infectious Diseases Society of America (IDSA) guideline, recommend rifampin with a fluoroquinolone companion (Osmon et al., 2013; Berbari et al., 2020). First, rifamycins are the drug of choice for persistent bacterial infections such as PJI (Robertson et al., 2008). Rifampin intervenes critical gene transcription process and prevents spread to deep-seated infection sites, crafting a clinical success against slow-growing and nonreplicating metabolic stages of bacteria (Robertson et al., 2008; Ma and Lynch, 2016). Second, the formation of bacterial biofilms, a polysaccharide glycocalyx that provides a mechanical shield from antibiotics and immune system of the host, over the prosthesis is a central mechanism in the pathogenesis of PJI (Taha et al., 2018). According to various *in vitro* and/or *in vivo* animal model studies, rifampin diffuses well into biofilm and produces effective killing against biofilm-associated bacteria (Sanchez et al., 2015). Third, a point mutation in the *rpoB* gene that encodes the β-subunit of RNA polymerase confers a high resistance to rifampin (Aubry-Damon et al., 1998; Silver, 2011), and a companion drug may be needed to slow down the development of resistance. In this context, *in vivo* and clinical data indicated fluoroquinolones as the best concomitant drugs with rifampin (Wells et al., 2018). However, the *in vitro* antagonism observed between rifampin and the fluoroquinolone class (Murillo et al., 2008) and the need to take rifampin orally and fluoroquinolones intravenously during the initial phase of the treatment regimen are major limitations for clinical use of this combination (Ma and Lynch, 2016). Rifampin inhibits bacterial RNA synthesis which may weaken the bactericidal effect of fluoroquinolones by impeding the supercoiling of DNA (Zimmerli and Sendi, 2019). To address these, a resolution by introducing a covalently linked hybrid of rifampin and a fluoroquinolone pharmacophore was proposed. Furthermore, fluoroquinolones are one of the most widely used classes in the antibiotic hybrid paradigm (Pham et al., 2019). A dual mechanism against topoisomerase IV and DNA gyrase, which may compensate for any interference shadowed by steric hindrance from a linker (Pokrovskaya and Baasov, 2010) or a second component with suitable attachment sites for bulky partners and stability under different synthetic conditions, are the arguments put forward for the fluoroquinolones (Fedorowicz and Sączewski, 2018).

**FIGURE 3:**
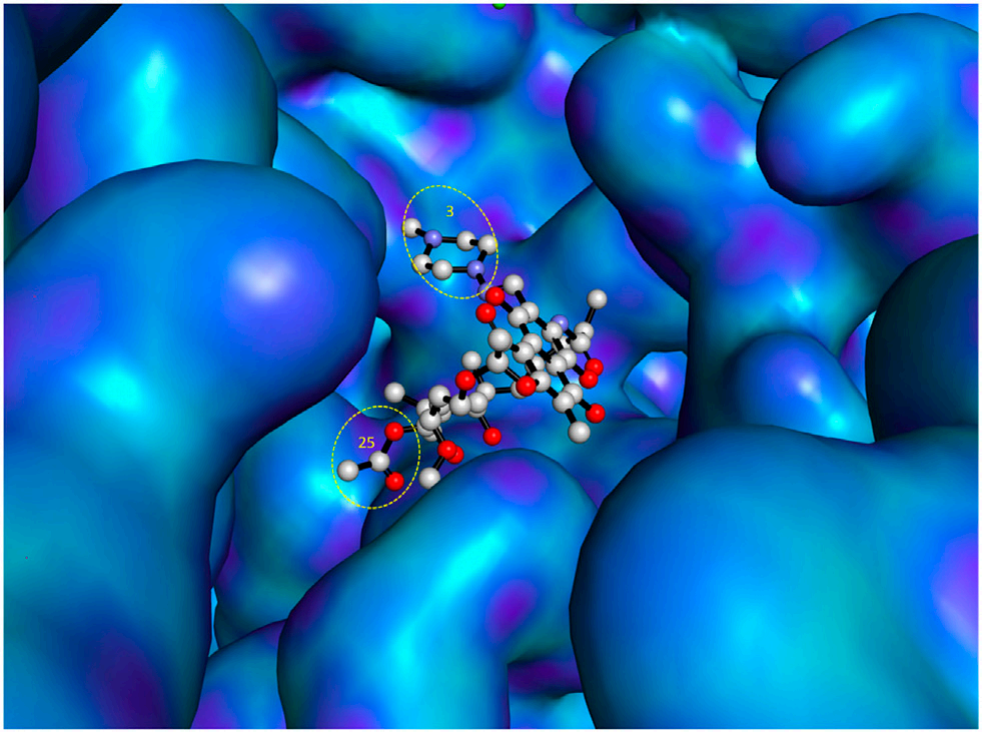
Most accessible positions for conjugation in the chemical structure of rifampin. The figure was generated from the crystal structure (Pdb 5UAL) (Molodtsov et al., 2017) using PyMOL (Delano, 2002).

As previously reported (Ma and Lynch, 2016), open spaces and hence convenient positions for attachments to the C-3 and C-25 positions of rifampin were identified from a co-crystallized structure of rifampin and RNA polymerase. This is further illustrated in Figure 3 using a recently reported co-crystal structure (Molodtsov et al., 2017). Structure–activity relationships (SARs) from a series of spirorifamycins disclosed that a heterocycle fused with both C-3 and adjacent C-4 of rifamycin can serve as an attachment site for the second partner (Kim et al., 2007). The carbonyl group at C-11 can be used as a conjugation site. Functionalization of the C-11 carbonyl to oxime led to a series of novel 11-deoxy-11-hydroxyiminorifamycin derivatives, with better or equivalent activity against the RNA polymerase of *S. aureus* (Li et al., 2007). Similarly, the C-25 position can be used for attachment of a companion after replacement of the acetyl group with a carbamate. A series of C-25 carbamate rifamycins showed improved antimycobacterial activity and absence of inactivation through ribosylation of C-23 alcohol by ADP-ribosyl transferase of *Mycobacterium smegmatis* (Combrink et al., 2007).

**FIGURE 4:**
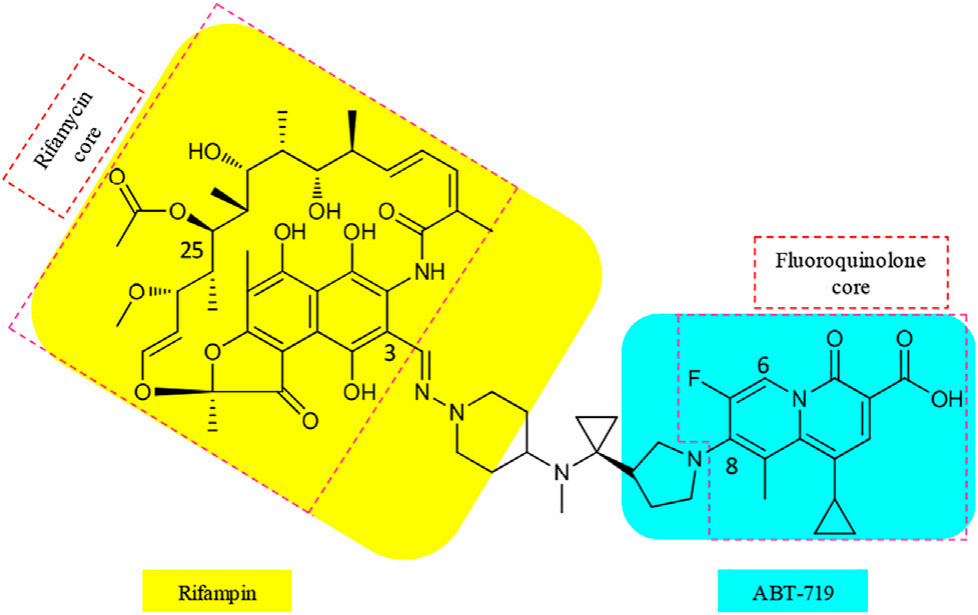
Cores and linker in the structure of TNP-2092 (**15**).

Various matching partners of rifamycins have been explored, with rifamycin–quinolones being the most studied (Ma and Lynch, 2016). SARs from approximately 300 rifamycin–quinolone hybrids revealed that the linker significantly influenced biological activity (Robertson et al., 2008; Klahn and Bronstrup, 2017). Partnering a rifamycin nucleus with a quinazolinone, a bioisostere of quinolone, provided the activity-leading conjugate, called CBR-2092 or TNP-2092 (**15**) (Figure 4, Ma and Lynch, 2016). Furthermore, the quinazolinone core possesses activity against the ParCS80F variant of topoisomerase IV, the activity that is not retained by ciprofloxacin. This provides an explanation for the activity of **15** against bacterial isolates that are resistant to ciprofloxacin alone or in combination with rifampin (Fisher et al., 2020). In **15**, the C-3 of rifamycin and the C-8 of quinazolinone pharmacophores are bound by a linker that resembles a covalently bound side chain of rifampin and ABT-719 (Ma and Lynch, 2016). The optimal linker in **15** also clamps the binding of rifamycin and quinazolinone motifs to their respective binding sites (Ma and Lynch, 2016).

TNP-2092 was superior to rifampin plus ciprofloxacin in several facets, a credence to hybrid pharmaceuticals in the antibacterial pipeline. 1) In *S. aureus* and coagulase-negative *Staphylococci* (coNS), the main causative agents and major elements of treatment failure in PJI, **15** has multi-targeted activities against RNA polymerase, DNA gyrase, and topoisomerase IV (Robertson et al., 2008; Ma and Lynch, 2016). 2) TNP-2092 is not a substrate for efflux pumps such as NorA or MepA and therefore retains activity against resistance mediated by mutations of the efflux system. In addition, the lack of efflux effectively traps **15** within bacteria (Ma and Lynch, 2016). Almost half of fluoroquinolone-resistant strains have enhanced expression of *norA* or *mepA* gene (Robertson et al., 2008). 3) TNP-2092 appears to overcome the antagonism between rifampin and ciprofloxacin (Murillo et al., 2008); in a minimum biofilm bactericidal concentration (MBBC) evaluation, ciprofloxacin plus rifampin exhibited MBBC levels higher than the MBBC values of ciprofloxacin alone in 11 of 40 *S. aureus* and in zero of 40 *S. epidermidis* isolates (Fisher et al., 2020). 4) TNP-2092 showed strong activity against pathogens resistant to its constituents. The difference in MIC values of **15** between the wild-type (CB190) and rifamycin-resistant *S. aureus* (CB370, *rpoB* ^H481Y^) was smaller, unlike 31,250 folds for rifampin. Compound **15** exhibited no activity difference between CB190 and fluoroquinolone-resistant *S. aureus* (CB814, *gyrA* ^S84L^ *ParC* ^S80F^), while nearly 67-fold difference was observed for ciprofloxacin (Robertson et al., 2008). Against rifampin-resistant *S. epidermidis* strains, IDRL-10005 (*rpoB* ^D471E^ *rpoB* ^I527Y^) and IDRL-10692 (*rpoB* ^S486F^), on the other hand, **15** had MIC values of 0.06 and 0.125 μg/ml, respectively; in contrast, rifampin had high MIC values of ≥4 μg/ml, and ciprofloxacin had MIC values of 8 and 1 μg/ml, respectively (Fisher et al., 2020). 5) Safety concerns related to constituent elements such as hERG inhibition and induction of CYP3A4 isoenzyme were not observed in **15** (Ma and Lynch, 2016).

Rifaximin, a member of the rifamycin family and a nonsystemic antibiotic, has been approved for the treatments of travelers’ diarrhea, hepatic encephalopathy, and irritable bowel syndrome without constipation (Lexicomp, 2021). Its clinical benefits were thought to be mediated through altered gastrointestinal (GI) microbiota or dysbiosis (Chey et al., 2020). Antibacterial evaluations of **15** against a representative panel of GI bacteria revealed similarities with rifaximin (Yuan et al., 2020). Interestingly, **15** was more active against Gram-negative bacteria such as *E. coli* and *A. baumannii*, which were resistant to rifampin (Ma and Lynch, 2016). The low oral bioavailability of **15**, 1.81% in rats and 0.315% in dogs, can offer additional benefits for local treatment of intestinal diseases (Yuan et al., 2020). The main strategy for the treatment of hepatic encephalopathy, which is closely related to hyperammonemia, is to regulate urease-producing bacteria located in the gut (Liu et al., 2018). Against a panel of urease-producing strains, **15** was more active than rifampin against Gram-negative bacteria such as *Helicobacter pylori* (*H. pylori*) and *Salmonella* strains but lower activity against beneficial commensals such as *B. infantis* and *B. bifidum* (Yuan et al., 2020). TNP-2092 appears to be an attractive candidate for the treatment of the urease-producing *C. difficile*. Compared to the standard treatments of *C. difficile* infections (CDIs), vancomycin and metronidazole, **15** showed superior activity against various isolates of *C. difficile* (Yuan et al., 2020). In addition, following 7 days’ treatment with **15**, the changes in the percentage of intestinal microbiota were temporary and generally returned to pretreatment levels. This explains the nonrecurrence of CDIs after treatment with **15** (Yuan et al., 2020). The standard treatments interfere with the intestinal microbiota and are the leading factors for the recurrence of CDIs (Tsutsumi et al., 2014).

The phase 1 study of **15** on tissue distribution, PK, safety, and tolerability in participants undergoing primary total hip replacement or knee arthroplasty is being planned, but participants have not been recruited yet (NCT04294862, 2021). Nevertheless, the phase 2 study of **15** for the treatment of ABSSSIs was completed in September 2020 (NCT03964493, 2019). TenNor Therapeutics presented “topline” phase 2 results of **15** for the treatment of ABSSSIs at the 2020 China Biomed Innovation and Investment Conference (CBIIC) (TenNor, 2020b). In 2020, the FDA has granted an orphan drug status for the IV use of **15** for the treatment of PJI (PR Newswire, 2020).

### Synthesis of TNP-2092

**SCHEME 2:**
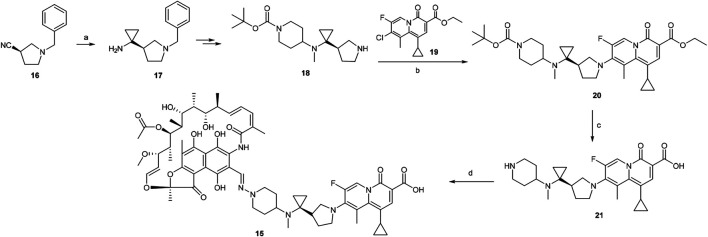
Synthesis of rifamycin hybrid **15** (Ma and Lynch, 2016). **(a)** Ti(O-*i*Pr)_4_, EtMgBr, Et_2_O/THF, −78°C, 20 min, then Et_2_O/BF_3_, rt, 2 h. **(b)** NaHCO_3_, acetonitrile, reflux, 5 h. **(c)** i. LiOH, ethanol, 60°C, 1 h; ii. TFA, dichloromethane, 0°C to rt, 1 h. **(d)** i. NaOH, H_2_N–OSO_3_H, 0°C, 1 h; ii. 3-formyl rifamycin, MeOH/THF, rt, 30 min.

A scheme suitable for kilogram quantity synthesis of **15** was reported (Ma and Lynch, 2016). The scheme involved an initial five-step synthesis of the linker intermediate **18**, which was subsequently coupled with the fluoroquinolone core **19** under reflux in acetonitrile, to give **20**. The fluoroquinolone-linker motif **20** was further processed *via* hydrolysis of ethyl ester and Boc deprotection to afford **21** using LiOH and TFA, respectively. Eventually, following the conversion of the piperidine moiety in **21** to a hydrazine, **15** was produced by coupling the hydrazine variant of **21** with 3-formyl rifamycin (Ma and Lynch, 2016; Scheme 2).

**FIGURE 5:**
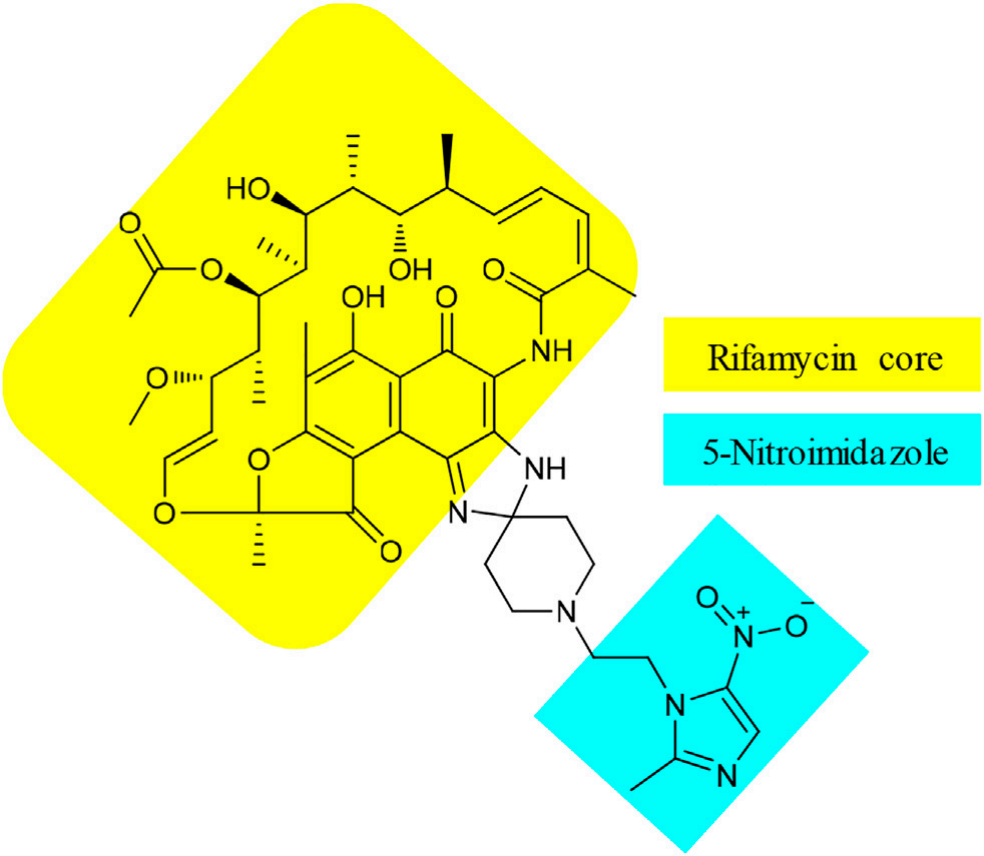
Chemical structure of TNP-2198 (**22**).

Besides **15**, TenNor Therapeutics is also developing a conjugate of rifamycin and metronidazole (Figure 5), also known as TNP-2198 (**22**). Metronidazole is used to treat infections caused by anaerobic bacteria and is the first-line treatment for bacterial vaginosis (BV) (Jones, 2019). However, the effectiveness of metronidazole against *Gardnerella vaginalis* (GV), the leading cause of BV, is associated with 58% recurrence (Bradshaw et al., 2012). The underlying factor for treatment failure is resistance by biofilms that form a protective shield to reduce the penetration of metronidazole (Ma et al., 2020). Leaving the essential nitroimidazole moiety intact, conjugates of metronidazole (Patel et al., 2021), *via N* ^1^-alkyl or 2-methyl linker or both, with different structural nuclei such as thiomorpholine-1,1-dioxide, have resulted in hybrids with superior activity against metronidazole sensitive as well as resistant *H. pylori* strains (Rossi and Ciofalo, 2020). Accordingly, the pharmacophore of metronidazole, a 5-nitroimidazole motif, was conjugated with rifamycin, an established agent against biofilm-forming pathogens. The resulting **22** showed strong synergistic property against GV (Ma et al., 2020). The MIC value of 0.004 μg/ml of **22** against GV was significantly lower than those of its constituents, with MICs of 4 and 0.5 μg/ml for metronidazole and rifampicin, respectively (Ma et al., 2020). In addition, the spectrum activity of **22** was not limited to GV but also active against other pathogens that can cause BV and other anaerobic bacteria. Apart from a few BV-causing bacteria where equivalent activity was displayed, the MIC values of **22** were 100 to 1,000 folds lower than those of metronidazole (Ma et al., 2020). Very recently, TenNor Therapeutics initiated a phase 1b/2a clinical development of **22** for the treatment of *H. pylori* infection (TenNor, 2020a). Additional clinical development status of **22** is listed in Table 1.

**TABLE 1:** Overview and key parameters of clinical macrocycle–antibiotic hybrids.

Macrocycle hybrid	Molecular weight (Dalton)	miLogP	tPSA	Select MIC_90_ (µg/ml)	Microorganism	*In vivo* ED_50_ (mg/kg)	Half-life (h)	Company	RoA	Clinical development status
TD-1792^a^	1,984.28	−4.68	673.39	0.03	MRSA, hVISA	0.19	1.7	Theravance biopharma/R-pharm	IV	Phase 2 completed in 2007 NCT00442832, (2006); phase 3 registered in Russia WHO (2019a)
TD-1607^b^	2,170.49	−4.98	751.81	0.008–0.06	MRSA	0.11	N/A	Theravance biopharma/R-pharm	IV	Phase 1 completed in 2014 NCT01949103, (2013); phase 1b completed in 2013 NCT01791049, (2013)
TNP-2092^c^	1,205.39	5.85	282.18	0.015	MSSA, MRSA	1.4–3.8	0.4–4.1	TenNor therapeutics	IV	Phase 1 for PJI (not yet recruiting) NCT04294862, (2021); phase 2 for ABSSSIs completed NCT03964493, (2019)
									PO	Phase 2 ongoing in China
									Topical	Preclinical development
TNP-2198^d^	944.05	2.70	269.21	0.004	GV	N/A	N/A	TenNor therapeutics	PO	Phase 1b/2a ongoing in China
DSTA4637S^e^	149 kDa (DSTA4637A TAb); 927.06 (dmDNA31)	4.30 (dmDNA31)	230.67 (dmDNA31)	0.004 µM (dmDNA31)	MRSA (USA300)	N/A	16.5–21.5 days	Genentech, Inc./Roche	IV	Phase 1 completed in 2016 NCT02596399, (2015)
										Phase 1b completed in 2020 NCT03162250, (2017)

MiLogP: the logP prediction developed at Molinspiration. tPSA: topological polar surface area. Both miLogP and tPSA values were calculated using Molinspiration cheminformatics (https://www.molinspiration.com/). RoA: route of administration; N/A: not available.

a MIC value (Blais et al., 2012); in vivo ED_50_ value (Long et al., 2008a); terminal half-life in mice (Hegde et al., 2012).

b MIC value (Sader et al., 2014); in vivo ED_50_ value (Long et al., 2008a).

c MIC value, in vivo ED_50_, and plasma half-life (Ma and Lynch, 2016); IV for medical device associated bacterial biofilm infections, PO for hepatic encephalopathy and irritable bowel symptom diarrhea, topical for superbugs and diabetic foot infection (TenNor Therapeutics, 2021).

d MIC value (Ma et al., 2020); PO for H. pylori and other anaerobic bacterial infections (TenNor Therapeutics, 2021).

e MIC value (Lehar et al., 2015); mean half-life of DSTA4637S TAb from phase 1 clinical trial (Peck et al., 2019).

**FIGURE 6:**
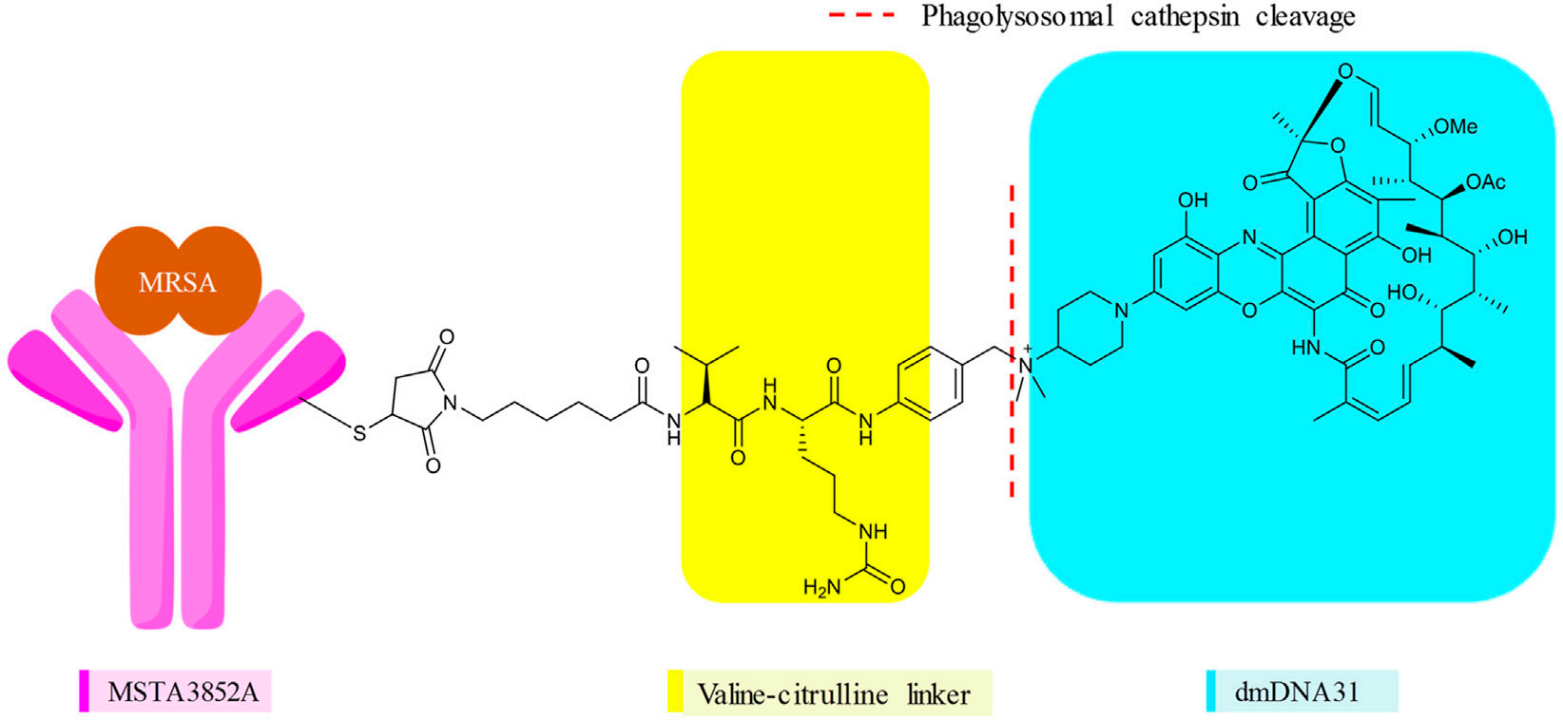
Monoclonal antibody, linker, and the antibiotic of DSTA4637S (**23**).

Antibody–antibiotic conjugation (AAC), in addition to the antibiotic-antibiotic hybrids mentioned above, is practiced for the conjugation of rifamycins. The antibody–drug conjugate (ADC) is mainly used in cancer immunotherapy, to selectively deliver a cytotoxic warhead using a labeling antibody bound *via* a linker (Beck et al., 2017). In 2015, the extension of ADC to the treatment of bacterial infections caused by *S. aureus* was first coined by Lehar et al. (2015). The infamous *S. aureus* is able to survive the wrath of antibiotics by internalizing into phagocytes (Fraunholz and Sinha, 2012). This intracellular reservoir has enabled long-term colonization of a host and promoted development of resistance, which explains the recurrences associated with invasive *S. aureus* infections (Lehar et al., 2015; Peyrusson et al., 2020). Despite available appropriate treatments, *S. aureus* remains a leading cause of death related to bacterial infections, with mortality rates of around 20–30% (Yilmaz et al., 2016). A new strategy capable of effectively eliminating the responsible intracellular foci of *S. aureus* was needed. As such, a novel AAC platform, THIOMAB™ antibody–antibiotic conjugate (TAC), was utilized to target the intracellular *S. aureus*. DSTA4637S (**23**) (Figure 6) is a novel conjugate of an antibiotic with an anti–*S. aureus* antibody, which represents the first AAC in this class under clinical development (Lehar et al., 2015; Mariathasan and Tan, 2017; Poreba, 2020). Mechanistically, the large-sized **23** cannot diffuse into mammalian cells. In systemic circulation and tissues, the antibody directs the binding of the conjugate to *S. aureus*, enabling the uptake of appended bacteria into phagocytes *via* opsonization (Cai et al., 2020). The linker is cleaved by cathepsins inside the phagolysosome to release the active antibiotic that can eliminate the conjugate bound and other existing bacteria (Lehar et al., 2015; Cai et al., 2020).

In DSTA4637S, an artificially engineered monoclonal antibody MSTA3852A against *S. aureus* from human immunoglobulin (IgG1) and 4-dimethylaminopiperidino-hydroxybenzoxazino rifamycin, dmDNA31 (rifalogue), are linked by a protease cleavable valine–citrulline (vc) linker (Lehar et al., 2015; Poreba, 2020). MSTA3852A was selected from >40 anti–*S. aureus* antibodies that originated from the blood of patients recovering from different *S. aureus* infections. The highest level of binding by those antibodies was directed against a major component in the cell wall of *S. aureus*, the wall-teichoic acids (WTAs) (Lehar et al., 2015; Deng et al., 2019).

**FIGURE 7:**
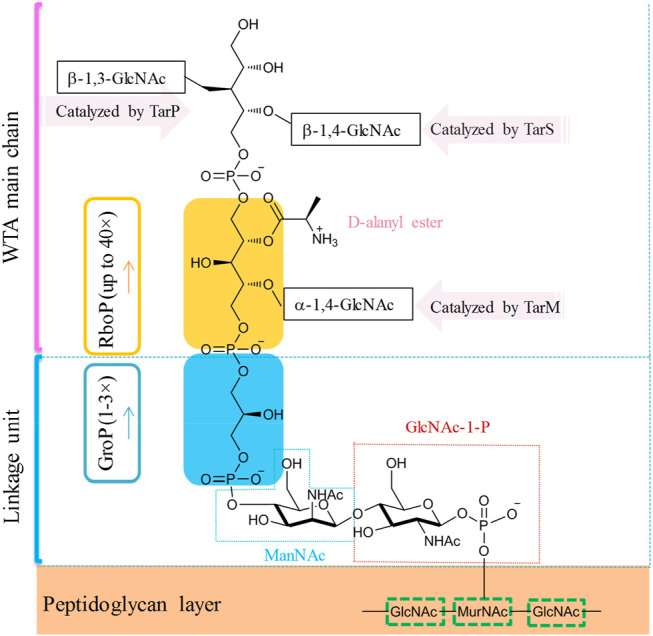
Simplified molecular structure of the WTA of common *S. aureus.*

In the majority of *S. aureus* lineages, WTA is composed of up to 40 repeating units of ribitol phosphate (RboP) that are covalently linked to the *N*-acetylmuramic acid (MurNAc) residue of the peptidoglycan layer by a short polysaccharide anchor unit (Figure 7, Weidenmaier and Peschel, 2008; Fong et al., 2018). The anchor unit is composed of glycerol-phosphate (GroP) units (1–3×), *N*-acetyl-d-mannosamine (ManNAc), and *N*-acetyl-d-glucosamine-1-phosphate (GlcNAc-1-P) (Winstel et al., 2014). RboP can be modified with α- or β-*O*-linked *N*-acetyl-d-glucosamine (α/β-*O*-GlcNAc) residues and d-alanine. Those modifications were reported to be essential for developing resistance to methicillin and cationic antimicrobial peptides (Brown et al., 2012). In MRSA strains, modifications with β-1,4-GlcNAc and α-1,3-GlcNAc are catalyzed by glycotransferases TarS and TarM, respectively (Brown et al., 2012; van Dalen et al., 2019). Recently, Gerlach et al. have identified an alternative glycotransferase for MRSA, TarP, which catalyzes the glycosylation of β-1,3-GlcNAc on WTA (Gerlach et al., 2018). This alternative glycan modification was poorly immunogenic and sabotaged the recognition by host antibodies (Gerlach et al., 2018). WTAs are promising antigens being pursued for the development of novel vaccines against MRSA (van Dalen et al., 2019).

**FIGURE 8:**
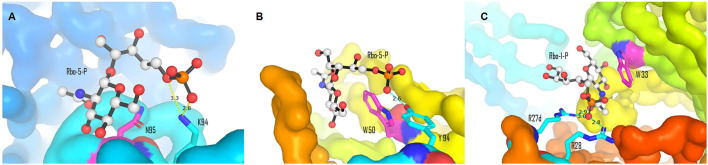
Binding interactions between the β-WTA mimic and anti–*S. aureus* antibodies. **(A)** Stacking and ionic interactions between antibody 4462 and Rbo-5-P (Pdb 6DWI). **(B)** Interactions between antibody 6078 and Rbo-5-P (Pdb 6DW2). **(C)** Arginine “tweezers” motif (Pdb 5D6C). Pictures **(A)**–**(C)** were generated from reported Pdb files (Lehar et al., 2015; Fong et al., 2018) using PyMOL (Delano, 2002).

The majority of the host anti–*S. aureus* antibodies target β-1,4-GlcNAc, followed by β-1,3-GlcNAc, and few antibodies are reactive to α-1,4-GlcNAc, albeit the underlying reason is not fully understood (van Dalen et al., 2020). The binding pattern between antibodies and β-WTA was then characterized by co-crystallizing the Fab fragment of anti–β-WTA antibodies with a minimal synthetic repeat of β-WTA, a β-glycosidic bonded unit between ribitol-1-phosphate (Rbo-1-P) or ribitol-5-phosphate (Rbo-5-P) and GlcNAc (Lehar et al., 2015; Fong et al., 2018). Antibodies displayed a conserved mechanism to specifically recognize the β-anomer mimic, albeit different residues were involved in different antibodies (Fong et al., 2018). In the co-crystal structures of antibody 4462 or 6078, for example, the pyranose ring of GlcNAc of the mimic was stacked against the amide chain of N95 or the indole side chain of W50, respectively (Figure 8, Fong et al., 2018). In antibody 4462, the C-5 phosphate of the WTA mimic formed ionic interactions with K94 (Figure 8A). Similarly, the interaction between the 5-phosphate and Y94 in antibody 6078 was indicated for selectivity to β-linked GlcNAc (Figure 8B, Fong et al., 2018). Lehar et al., using a co-crystal structure of antibody 4497 with 1-phosphoribitol β-WTA, also revealed an arginine “tweezers” motif by Arg27d and 28 that dictated the β-anomer–specific recognition *via* triangulation of the ribitol phosphodiester backbone in relation to the GlcNAc moiety (Figure 8C, Lehar et al., 2015).

Various features of rifalogue (dmDNA31) were considered in the selection of the payload for anti–*S. aureus* antibody 4497. dmDNA31 had an MIC value of 4 × 10^–9^ M against MRSA (USA300), with effective killing property against these well-recognized persister cells (Lehar et al., 2015). Persister cells are dormant, nonreplicating bacteria with a high level of tolerance to antibiotics. Nondividing MRSA isolates inside macrophages were effectively eliminated by dmDNA31 (Lehar et al., 2015). Moreover, dmDNA31 conjugate was inactive against extracellular bacteria, indicating the safety of the conjugate (Shopsin et al., 2016).

V112C mutagenesis of the antibody MSTA3852A, in which a valine in the light chain IgG1 is precisely replaced by a reactive cysteine residue, enables site-specific conjugation with a drug–antibody ratio (DAR) of two vc-dmDNA31 per antibody (Deng et al., 2019). Site-specific conjugation in ADC enables a direct and homogeneous conjugation of a drug (Zhou, 2017). Stochastic or random conjugation, on the other hand, often leads to heterogeneous products, with each subtype having different PK properties, efficacy, and safety profiles. In stochastic conjugates, there are also concerns about higher DAR, which are relatively more active *in vitro*, less tolerated, and exhibit rapid elimination than conjugates with lower DAR (Gauzy-Lazo et al., 2020). THIOMAB™ is one such site-specific conjugation technology that led to **23** (Lehar et al., 2015; Wang-Lin and Balthasar, 2018; Gauzy-Lazo et al., 2020). The linker in **23** will be cleaved within minutes following the entry of the opsonized *S. aureus*, and when tested inside human macrophages, epithelial and endothelial cells, AAC-opsonized MRSA isolates were killed in all cases (Lehar et al., 2015). In an intravenous mice infection model, the efficacy of **23** was superior than that of **1**. The efficacy of **1** declined when administered after 7–24 h of infections, suggesting the failure of **1** due to the internalization of MRSA into host cells (Lehar et al., 2015). After 24 h of infection, a single dose of TAC was effective and superior to the twice-daily dose of **1** (Lehar et al., 2015).

The preclinical PK properties of TAC **23** were profiled in mice, rats, and cynomolgus monkey (Zhou et al., 2016; Deng et al., 2019). The PK profiling of TAC involved the analysis of three analytes: TAC total antibody (TAb) which includes fully conjugated (DAR 2), partially conjugated (DAR 1), and unconjugated antibodies (Zhou et al., 2016). The PK properties of typical monoclonal antibody-based therapeutics, that is, short distribution, slow clearance, and long half-life, were demonstrated by DSTA4637A (the preclinical liquid formulation of **23**) in various *in vivo* animal studies (Zhou et al., 2016; Deng et al., 2019). In mice, the conjugation extended the half-life (∼3–4 h) of unconjugated dmDNA31 to 4 days in ac-dmDNA31. A similar clearance between **23** and the unconjugated antibody in mice indicated the conjugation of dmDNA31 had relatively little effect on the clearance of the TAb (Zhou et al., 2016). Comparable PK properties of DSTA4637A TAb and MSTA3852A were also found in rats and monkey (Deng et al., 2019). Three main reasons can explain the similar PK profiles between DSTA4637A and MSTA3852A. First, conjugates with a DAR of 2–4 generally have slower clearance rates and longer half-lives than conjugates with a higher DAR (Deng et al., 2019). Second, THIOMAB™ technology allows uniform conjugation on the engineered cysteine without disrupting the inter-disulfide bonds in the antibody (Sussman et al., 2018), and therefore minimal PK changes after conjugation. Third, drug loading and hydrophobicity are factors that cause significant changes in PK properties (Kamath and Iyer, 2016). The hydrophilic property of dmDNA31 is expected to slow down clearance and extend the half-life of **23** (Deng et al., 2019). A minimal physiologically based PK model in mice indicated a low level of drug interactions between DSTA4637A and cytochrome P450 substrates. This model of PK disposition also indicated the liver and spleen, where phagocytes are usually accumulated, may contain high concentrations of dmDNA31 (Wang-Lin et al., 2018).

Preclinical safety evaluations with high doses of **23**, up to 250 and 500 mg/kg in monkeys and rats, respectively, were well tolerated (Deng et al., 2019). In 2019, the results of the phase 1 investigations on the safety, PK, and immunogenicity in healthy volunteers were published, which showcased the favorable profiles of **23** for human use (NCT02596399, 2015; Peck et al., 2019). No serious adverse effects were observed in the dose range between 5 and 150 mg/kg; no clinically significant changes in laboratory or vital signs or antibody responses induced by **23** were observed (Peck et al., 2019). Therefore, further clinical development of **23** for *S. aureus* infections is expected following these favorable safety and PK profiles in human volunteers (Peck et al., 2019).

## Limitations and Ways Forward in Macrocycle-Antibiotic Hybrid Approach

The increase in molecular weight is a major limitation of the antibiotic hybrid approach. The resulting low oral bioavailability of such conjugates may impede oral dosage formulations for systemic applications (Gupta and Datta, 2019). However, this opens the door to selectivity and efficacy for local treatments of gut and liver diseases (Ma and Lynch, 2016). In addition, given that the structure of the outer membrane (OM) in the cell wall of Gram-negative bacteria is well designed for intrinsic resistance to various antibacterial agents, the high molecular weight of antibiotic hybrids is also detrimental to their activity against Gram-negative microorganisms. Moreover, the hydration sphere, created by hydrophilic carbohydrates, and the reduced fluidity caused by efficient packing of the lipid component restrict the passage of hydrophobic molecules across the OM (Domalaon et al., 2018). What remains is a passage through unspecific, barrel-shaped protein channels called porins. Nevertheless, only small molecules with a molecular weight of ≤600 g/mol are more favorable to pass the molecular sieve imposed by the porins (Vergalli et al., 2020). Fortunately, some antibiotics with a molecular weight of >600 g/mol, such as polymyxins, are able to pass through OM *via* various uptake mechanisms (e.g., self-promoted entry) (Moubareck, 2020). Agents of this nature are capable of disturbing the electrostatic interactions between the divalent cations such as Mg^2+^ or Ca^2+^ and phosphate groups in the lipid, thereby creating a passage through (Deris et al., 2014). Further clarifications of the structural or physicochemical criteria for self-promoted mechanism can extend the antibacterial spectrum of macrocycle-antibiotic hybrids against Gram-negative organisms (Domalaon et al., 2018).

Pioneered by cefiderocol, a cephalosporin siderophore antibiotic approved by the U.S. FDA in November 2019 (Lexicomp, 2021), antibiotic–siderophore conjugates represent an attractive antibiotic hybrid class by conjugating iron-chelating microbial siderophores with an antibiotic to facilitate uptake and antibacterial efficacy (Schalk, 2018; Negash et al., 2019). Specifically, a macrocycle siderophore hybrid, involving the conjugation of a macrocyclic antibiotic with a microbial siderophore or a siderophore mimetic, may overcome the penetration issue to pass through the OM of Gram-negative bacteria. Siderophores benefit bacteria by dissolving and importing iron (Ji et al., 2012). Conjugating with a siderophore can facilitate the entry of the corresponding hybrid through a ferri-siderophore uptake pathway that internalizes iron by an active transport mechanism, that is, a Trojan horse mechanism (Schalk, 2018). Similarly, a self-promoted passage through means of polymyxins can be exploited to force the entry of macrocycle polymyxin conjugates (Negash et al., 2019). Conjugation with polymyxins can not only extend activity against Gram-negative bacteria but also retain activity against resistance mechanism involving overexpression of efflux pumps as well (Negash et al., 2019). Various other hybrid chemical entities have also been reported, including polymyxin B3-tobramycin hybrids with *Pseudomonas eruginosa*–selective antibacterial activity and strong potentiation of rifampicin, minocycline, and vancomycin (Domalaon et al., 2017), azithromycin–benzoxaborole hybrid derivatives (Tevyashova et al., 2019), as well as antitubercular rifampicin and clofazimine hybrid (Saravanan et al., 2021). Very recently, design and synthesis of vitamin B12–antibiotic conjugates led to advanced candidates with >500-fold improved activity against Gram-negative bacteria including *E. coli*, relative to ampicillin, demonstrating that the vitamin B12 conjugate strategy is effective for enabling cellular uptake and antibiotic delivery, thus improving antibacterial efficacy (Zhao et al., 2020). Furthermore, among numerous elegant examples by the Schweizer group (Gorityala et al., 2016a; Gorityala et al., 2016b; Yang et al., 2017; Domalaon et al., 2019), the use of antibiotic hybrids as adjuvants such as nebramine-based hybrids (Yang et al., 2019) and lysine-tobramycin conjugates (Lyu et al., 2019) has potentiated the activities of current existing antibiotics including rifampicin and erythromycin, respectively. Similarly, rifamycin–tobramycin conjugate adjuvants were able to break intrinsic resistance of *Pseudomonas aeruginosa* to tetracyclines and chloramphenicol (Idowu et al., 2019).

Some key questions remain to be answered despite the progress and new advances of antibiotic hybrids to clinical development (Domalaon et al., 2018), highlighting the potential to burgeon the antibacterial pipeline. Structure-based drug design, exemplified by X-ray co-crystal studies and molecular modeling, is among techniques capable of ameliorating the way forward of antibiotic hybrids. For example, the co-crystal structure of rifampin with RNA polymerase has highlighted steric-free sites of attachments for conjugates, *vide supra* (Ma and Lynch, 2016; Molodtsov et al., 2017). In addition, TenNor Therapeutics very recently presented the outcome of their phase 2 clinical trial of TNP-2092 and disclosed the co-crystal structure of TNP-2092 bound with bacterial RNA polymerase, along with its interaction with DNA, at the 2020 CBIIC, which was held in Suzhou, China, in September 2020 (TenNor, 2020b). Computational strategies were also applied in the design of promising hybrids which are in the early stage of development. For example, in the design of macrocyclic peptide–peptoid hybrids, the crystal structure of the chemokine receptor CXCR4 was used as a template for the homology model of CXCR7 (Boehm et al., 2017). Besides identifying residues important for activity, binding poses from an induced fit docking also spotted spaces in the CXCR7 model that are not occupied by the macrocycle peptide (Boehm et al., 2017). Virtual tools also facilitated the design of VAN–nicin conjugates, by predicting the optimal length of the linker and suitable attachment sites (Arnusch et al., 2008). Nicin is an antimicrobial peptide that binds to lipid II and inhibits the transglycosylation step in the cell wall biosynthesis (Le et al., 2017). Although each has different modes of action, lipid II is targeted by both VAN and nicin. Predicting the optimal spacer and sites of connection was essential to place the constituting elements to their respective binding sites in lipid II (Olsufyeva and Tevyashova, 2017). Computationally guided design of VAN–nicin hybrids led to promising derivatives, with the most active hybrid displaying a 40-fold higher activity than VAN or nicin (Arnusch et al., 2008).

Besides activity profiling, virtual tools depending on prior knowledge or machine learning may enable prediction of specificity, solubility, permeability, and general toxicity of hybrids (Mulligan, 2020; Zin et al., 2020). Prediction of physical properties using molecular dynamics, a computer simulation of the movement of a molecule surrounded by water, may provide a more unbiased model than tools dependent on a database of a large number of compounds with known properties (Mulligan, 2020). Efficient and reliable computational tools for predicting physicochemical properties such as permeability can be essential in the design of macrocyclic hybrids. Macrocycles and their antibiotic hybrids/conjugates are generally considered poor drug-like compounds and often violate desirable parameters for orally bioavailable drug molecules (Lipinski et al., 1997; Zin et al., 2020). Diverse functionalization strategies of macrocycles can modulate physicochemical properties by changing the rigidity, conformation, and basicity of the macrocycle core and/or its side chain (Mallinson and Collins, 2012). In addition, the decrease in degree of freedom by macrocyclization may improve cell permeability of macrocycles (Dougherty et al., 2019). A recent study demonstrated modulation of scaffold rigidity to engineer favorable ADME properties in macrocyclic peptides (Furukawa et al., 2020). Finally, drug design strategies, such as *N*-methylation or replacing NH with sulfur (or vice versa), were found to improve pharmacological activity, solubility, and/or permeability of macrocycles (Mallinson and Collins, 2012; Liras and McClure, 2019; Stadelmann et al., 2020; Buckton et al., 2021). Extending studies of this nature to macrocyclic hybrids is expected to improve their oral bioavailability for systemic uses.
